# Supplementary material for: Defining the early stages of intestinal colonisation by whipworms
Source: Nat Commun. 2022 Apr 1;13:1725. doi: 10.1038/s41467-022-29334-0 (PMC8976045; doi:10.1038/s41467-022-29334-0)
Supplement: Supplementary file 1 — Supplementary Information [file 41467_2022_29334_MOESM1_ESM.pdf]

DEFINING THE EARLY STAGES OF INTESTINAL COLONISATION BY WHIPWORMS

SUPPLEMENTARY FIGURES

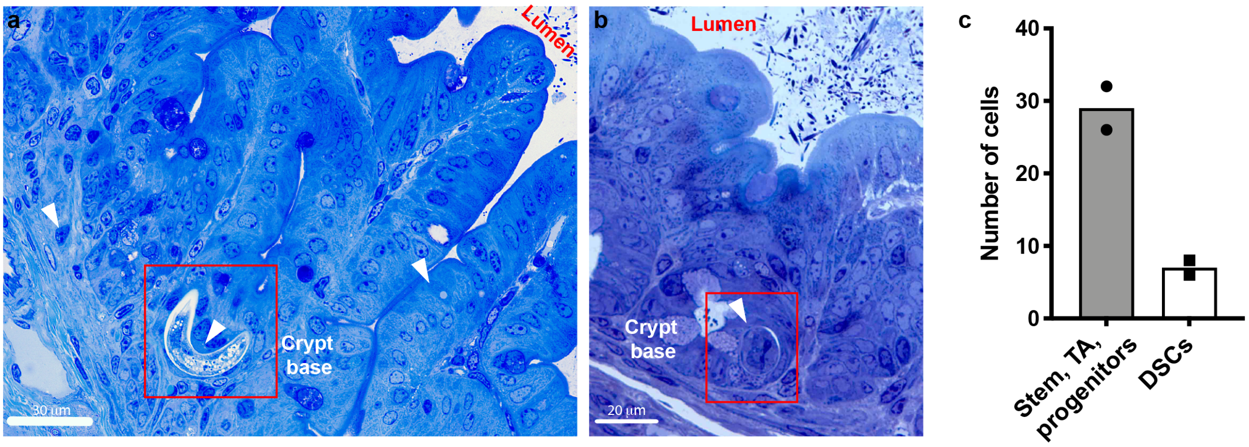

Supplementary Fig. 1. Whipworm L1 larvae become completely intracellular in syncytial tunnels at the bottom of the crypts of Lieberkühn.

a-b Images of toluidine blue-stained transverse sections from caecum of WT mice infected with *T. muris* at 3 h p.i., showing whipworm larvae (arrowhead) infecting cells at the base of crypts. Images are representative from 15 larvae found during the first 72 h of infection across two independent experiments with three mice per time point (3, 24, and 72 h p.i.) each. **c** Numbers of stem, TA, progenitor cells and DSCs infected by whipworm L1 larvae in syncytial tunnels *in vivo* (24 h p.i.). Counts on serial block face SEM images of two syncytial tunnels. Points are the counts for each individual worm. Source data are provided as a Source Data file.

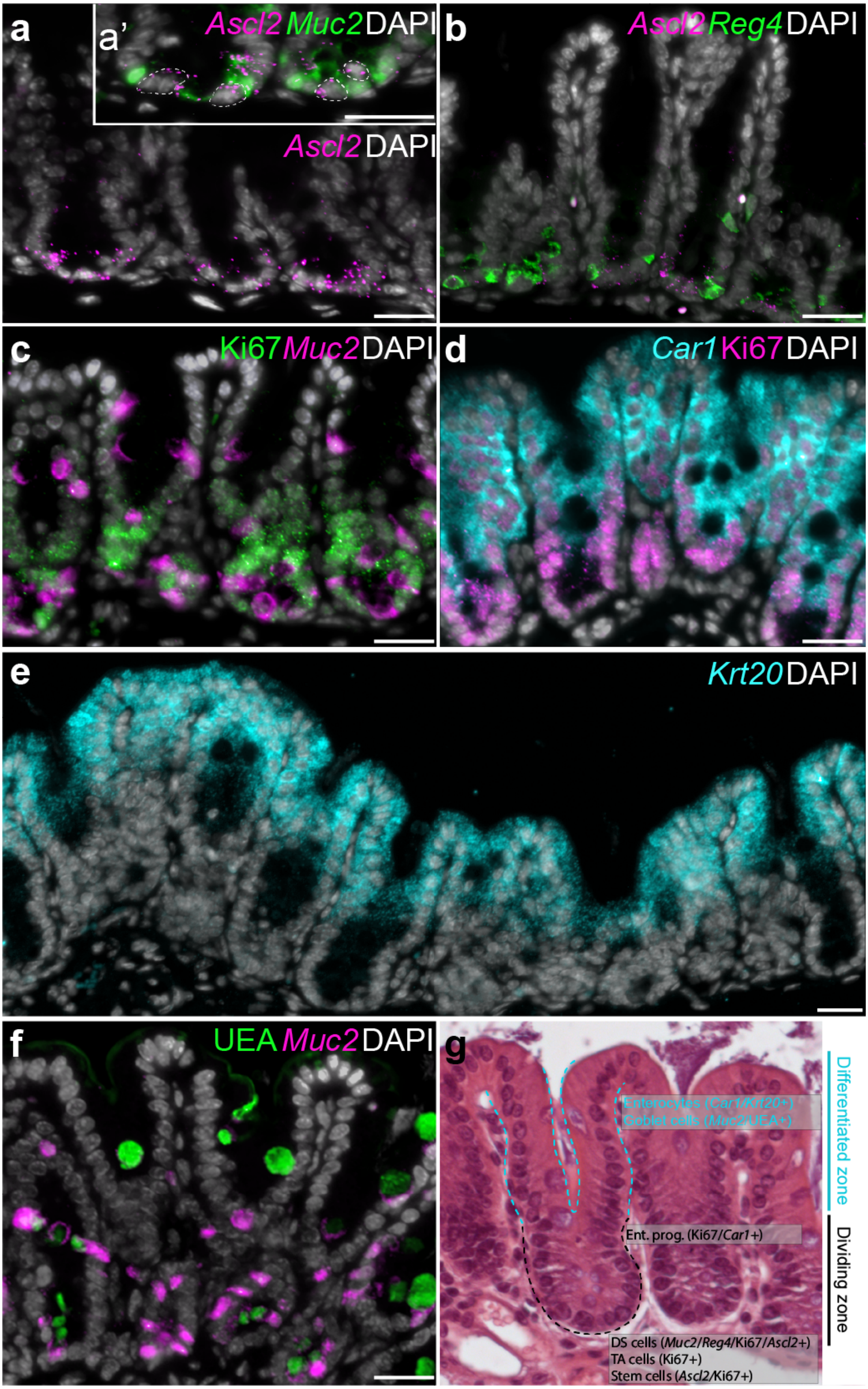
a
a'
Ascl2 Muc2 DAPI
Ascl2 DAPI
b
Ascl2 Reg4 DAPI
c
Ki67 Muc2 DAPI
d
Car1 Ki67 DAPI
e
Krt20 DAPI
f
UEA Muc2 DAPI
g
Enterocytes (Car1/Krt20+)
Goblet cells (Muc2/UEA+)
Ent. prog. (Ki67/Car1+)
DS cells (Muc2/Reg4/Ki67/Ascl2+)
TA cells (Ki67+)
Stem cells (Ascl2/Ki67+)
Differentiated zone
Dividing zone

Supplementary Fig. 2. Molecular characterisation of cell types within the mouse caecum. **a** *Ascl2* (magenta) is expressed by cells in the bottom of the caecal crypts. Within this region, **a'** *Ascl2* (magenta) transcripts co-localise with expression of *Muc2* (green) in DSCs, and are also detected within *Muc2*$^{-}$ cells (putative stem cells, indicated with dashed white outlines). **b** *Reg4* (green) is expressed in DSCs at the bottom of the caecal crypts. **c** Ki67 (green) is expressed broadly throughout the bottom of the caecal crypts, in a territory encompassing *Muc2*$^{+}$ (magenta) DSCs, as well as *Muc2*$^{-}$ cells (putative stem and TA cells). *Muc2* is also expressed in Ki67^{-} goblet cells. **d-e** Differentiated enterocytes within the caecum express *Car1* and *Krt20* (aqua), while enterocyte progenitors co-express *Car1* (aqua) and Ki67 (magenta). **f** *Muc2*$^{+}$ (magenta) DSCs and goblet cells also stain positively with the lectin UEA (green), but can be distinguished by location and morphology - i.e. DSCs are at the base of the crypt and are not morphologically distinguishable from other cell types within the base crypt by standard microscopy, while goblet cells are located at the top of the crypts and crypt table and are very large and round. **g** The caecal epithelium is therefore broadly organised into a Ki67^{+} "dividing zone" (dashed black outline), which includes stem, TA, DSCs and enterocyte progenitor cells, and a Ki67^{-} "differentiated zone" (dashed blue outline), which includes enterocytes and goblet cells. In **a** to **f**, in white DAPI stains nuclei. Images in **a** and **a'** are from different sections. Images are representative of sections from three mice. All scale bars = 15 μm**.**

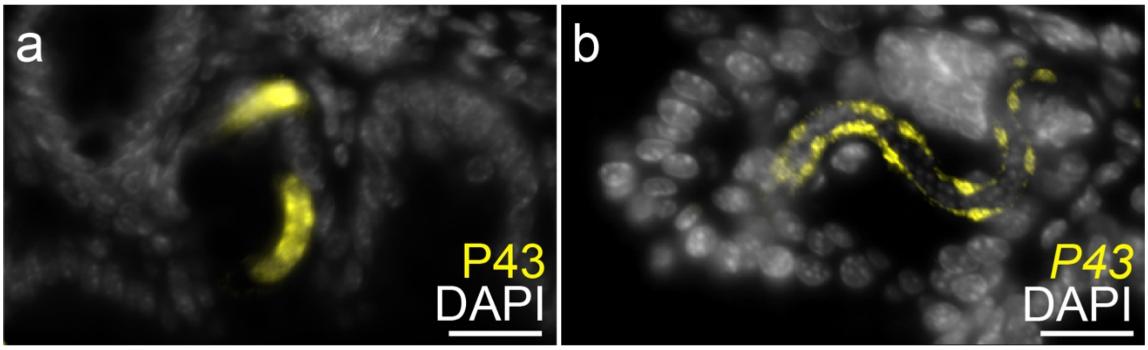

Supplementary Fig. 3. Detection of *T. muris* L1 larvae in the mouse caecum using IF and ISH HCR.

a Detection of *T. muris* p43 protein (by IF, in yellow) and **b** *p43* transcripts (by ISH HCR, in yellow) facilitated location of worms within infected caecal tissue. In white DAPI stains nuclei. Image on **a** is representative of sections from two mice (one at 24 and one at 72 h p.i.), in which 3-5 L1 larvae per mouse were imaged. Image on **b** is representative of sections from three mice at 72 h p.i., in which a total of 31 L1 larvae were imaged. All scale bars = 15 μm**.**

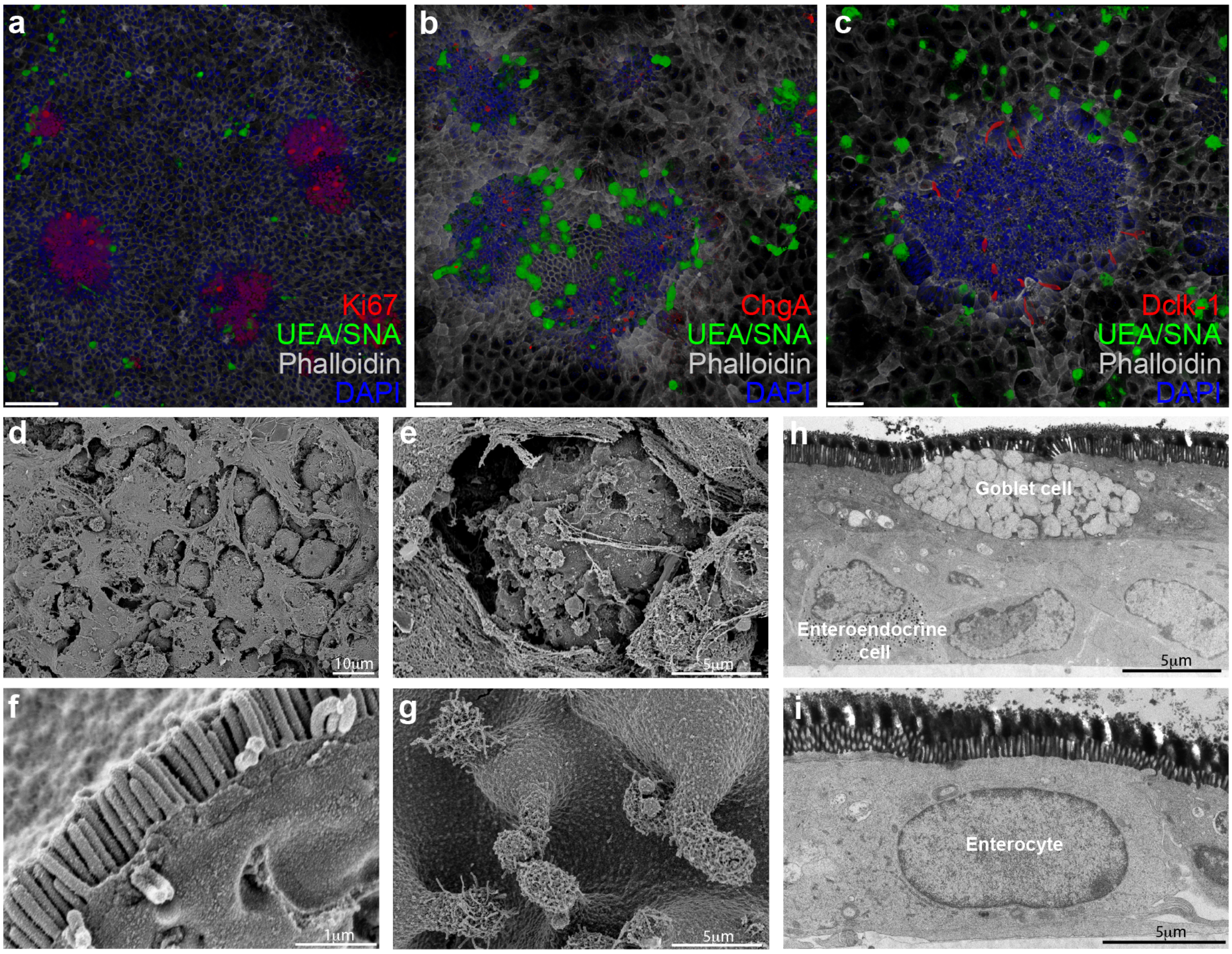

Supplementary Fig. 4. Characterisation of murine caecaloids grown in transwells.

Images of caecaloids grown and differentiated in transwells showing the presence of all IEC populations organized in structures that recapitulate caecal epithelia cell-type composition and crypt organisation. **a-c** Confocal IF microscopy with antibodies staining **(a)** Ki-67 (red), marker of proliferating cells; **(b)** chromogranin A (red) expressing enteroendocrine cells; and **(c)** Dclk-1 (red), marker of tuft cells. Lectins UEA and SNA (green) bind mucin glycans in goblet cells. DAPI (blue) stains nuclei and phalloidin (white) binds to F-actin. Scale bar 100 μm for a, 50 μm for b and c. **d-g** Scanning EM images showing **(d)** mucus and **(e)** goblet cells, **(f)** microvilli of enterocytes and **(g)** tuft cells. **h-i** Transmission EM showing **(h)** goblet and enteroendocrine cells and **(i)** enterocyte**.** Imaging experiments on caecaloids were done in triplicate across more than ten independent replicas using three caecaloid lines derived from three C57BL/6 mice.

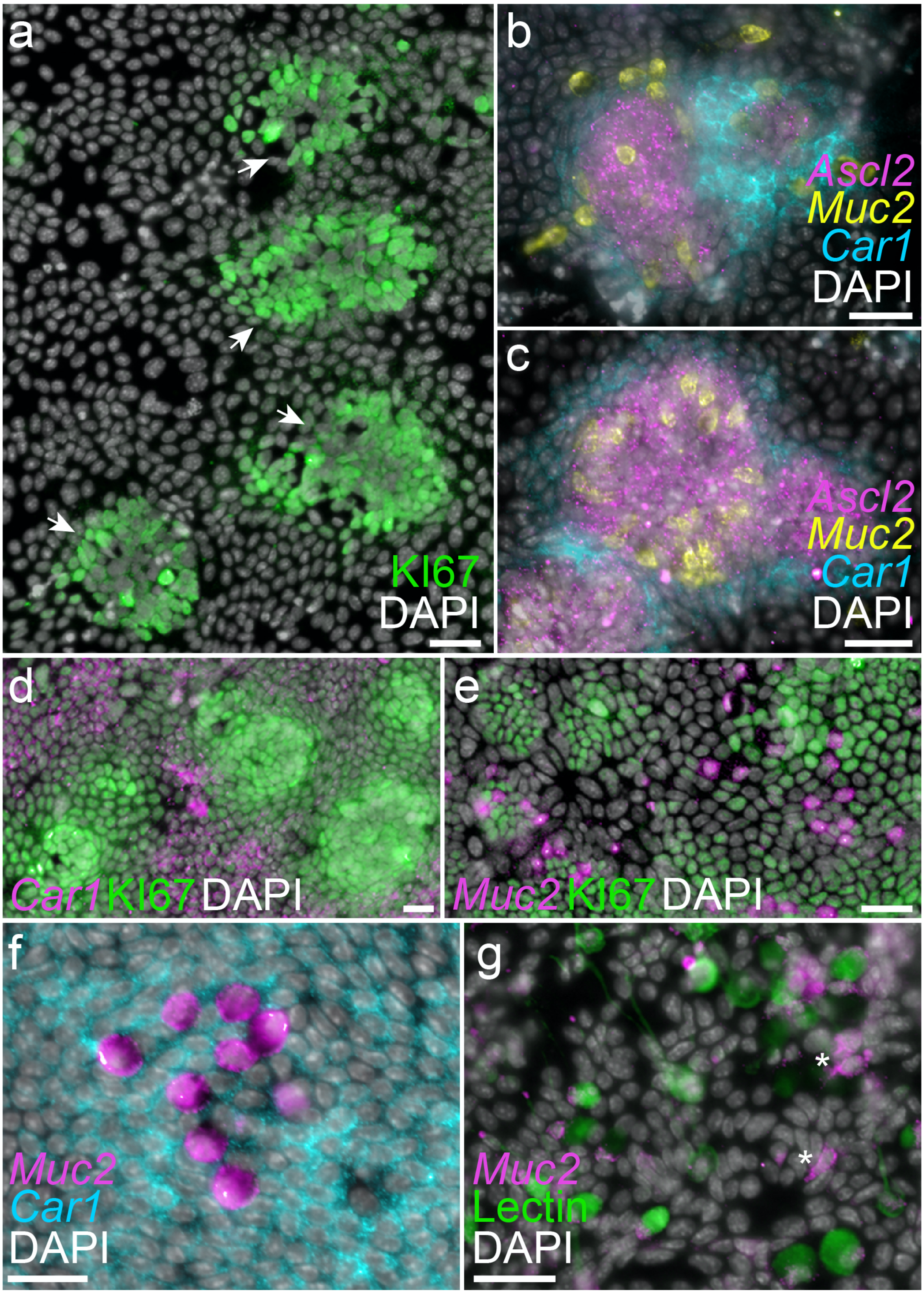

Supplementary Fig. 5. Molecular characterisation of cell types within murine caecaloids.

a Murine caecaloids contain clusters of mitotically active, Ki-67$^+$ (green) epithelial cells (white arrows). **b-c** These clusters contain *Ascl2*$^+$ (magenta) putative stem cells and *Muc2*$^+$ (yellow) DSCs, and are surrounded by *Car1*$^+$ (aqua) enterocyte progenitors/ enterocytes; thus, recreating the “dividing zone” at the bottom of the crypts in the caecum. **d** *Car1*$^+$ (magenta) enterocyte progenitors/ enterocytes adjacent to clusters of Ki-67$^+$ (green) cells. **e** *Muc2*$^+$ (magenta) DSCs and goblet cells

adjacent to clusters of Ki-67$^+$ (green) cells. Caecaloids also contain differentiated areas with **f** *Car1*$^+$ (aqua) enterocytes, and large, rounded *Muc2*$^+$ (magenta) goblet cells, **g** which also stain positively with the lectin UEA (green). *Muc2*$^+$ DSCs (*) are distinguishable from goblet cells based on morphology and location within the epithelial layer. In white DAPI stains nuclei. All scale bars = 15 μm. ISH by HCR imaging experiments on caecaloids were done in triplicate across two independent replicas using two caecaloid lines derived from two C57BL/6 mice.

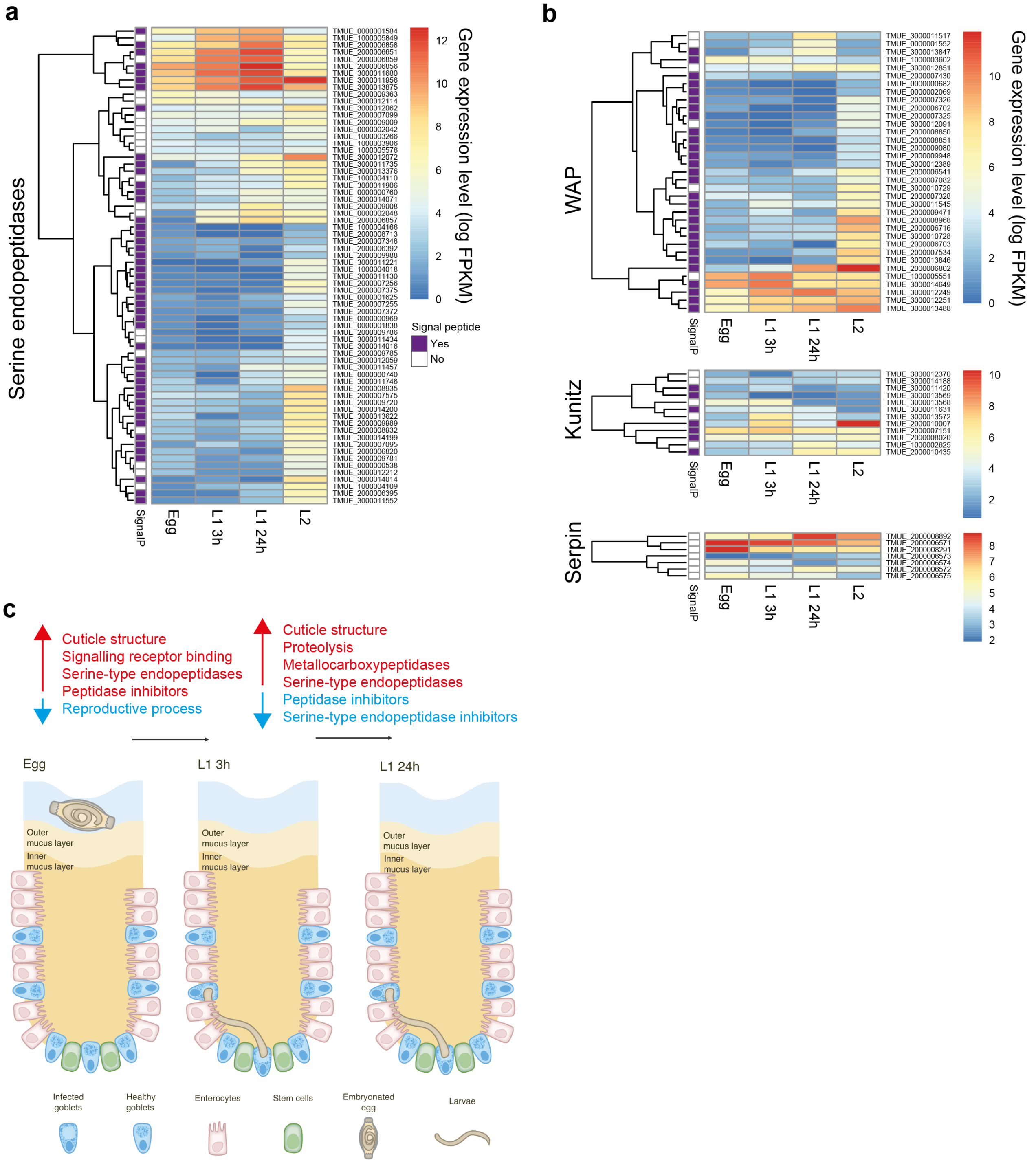

Supplementary Fig. 6. Expression of serine proteases and peptidase inhibitors by L1 larvae in early stages of infection.

Expression levels of **a** serine endopeptidases and **b** WAP, Kunitz and serpin peptidase inhibitors from *T. muris* are shown over early larval development as log-normalised Fragments Per Kilobase per Million mapped reads (FPKM). Only genes differentially expressed across the time course are shown. Larvae were recovered at each time point from two infected mice across three independent experiments for a total n=6. **c** A diagram of early larval development in a caecal crypt is annotated

with examples of GO terms enriched amongst genes with increasing transcript levels (red) or reduced transcript levels (blue) between stages.

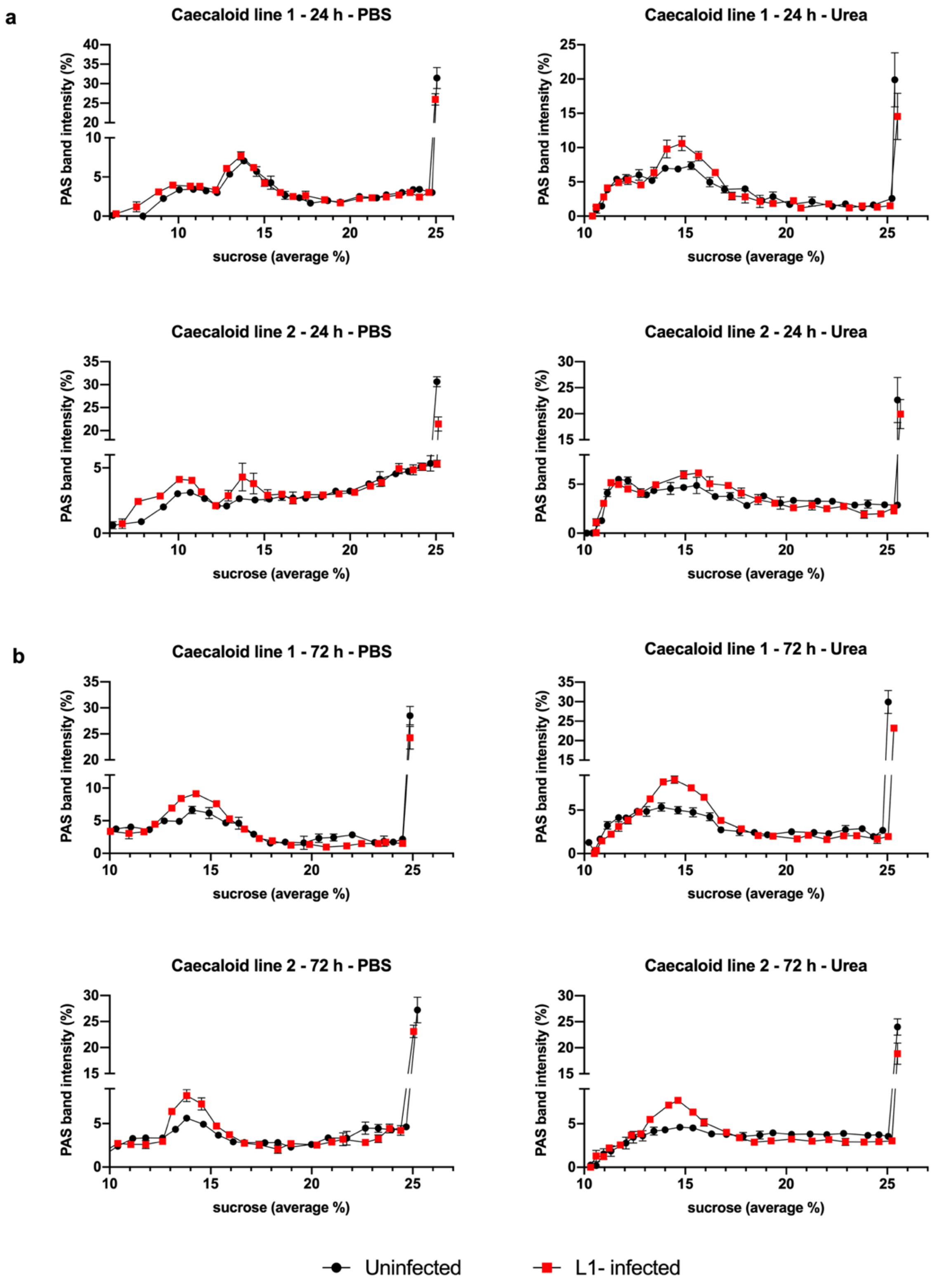

Supplementary Fig. 7. Mucus degradation by L1 larvae in early stages of infection.

Caecaloid mucus degradation by L1 larvae at **a** 24 and **b** 72 h p.i. Transwells were washed with PBS followed by 0.2 M urea in PBS to recover mucus. Washes were subjected to rate zonal centrifugation on linear 5-25% sucrose gradients. After centrifugation tubes were emptied from the top and the fractions were stained with PAS to detect the mucins. Data are shown as percentage of intensity.

Black circles represent uninfected caecaloids and red squares represent *T. muris* L1-infected caecaloids. Results are represented as the mean +/- SEM of 3 replicas of two caecaloid lines. Source data are provided as a Source Data file.

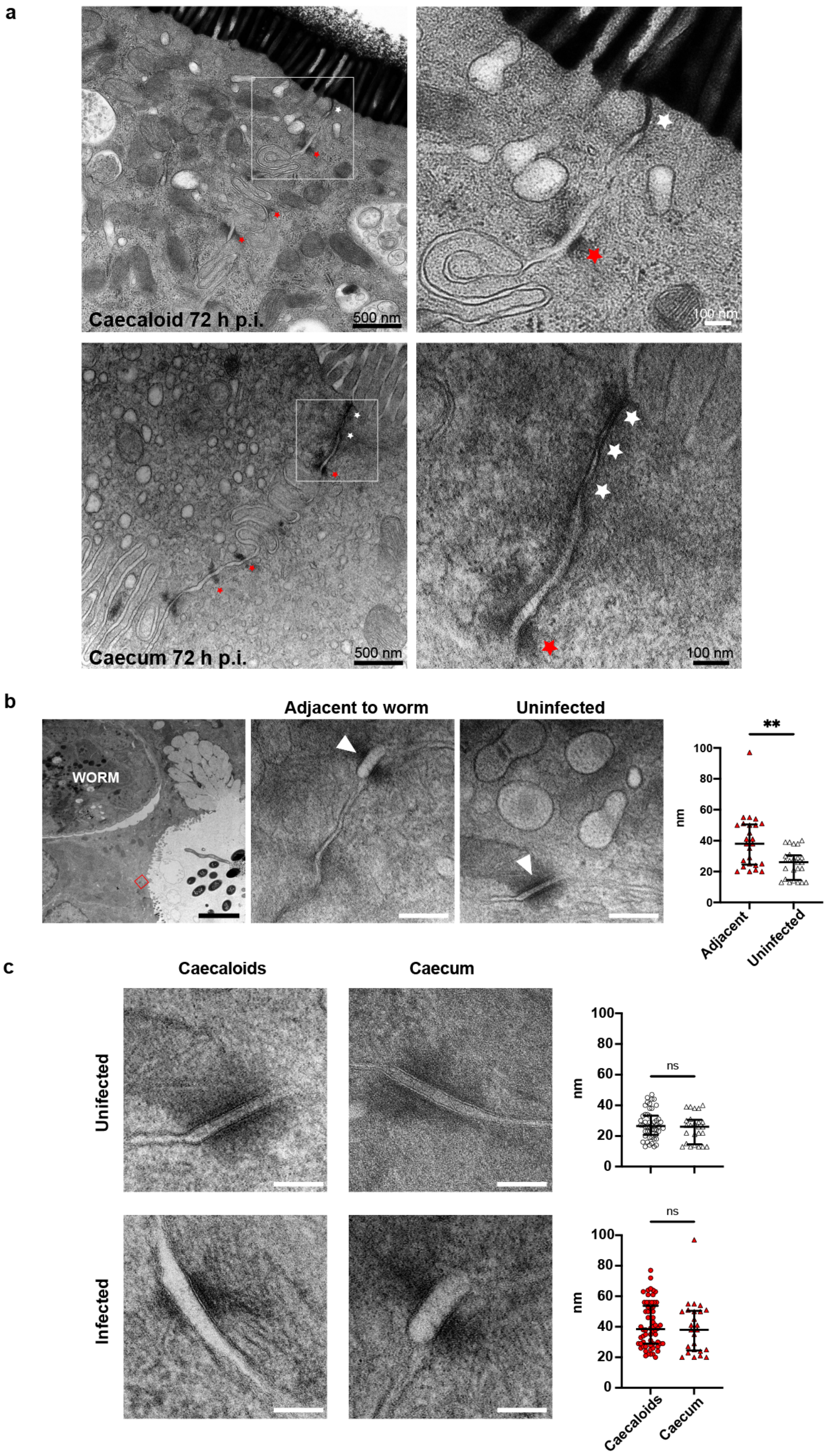
a
Caecaloid 72 h p.i.
500 nm
100 nm
Caecum 72 h p.i.
500 nm
100 nm
b
WORM
Adjacent to worm
Uninfected
**
100
80
60
40
20
0
nm
Adjacent
Uninfected
c
Caecaloids
Caecum
Unifected
Infected
100
80
60
40
20
0
nm
ns
ns
Caecaloids
Caecum

Supplementary Fig. 8. Perturbation of host cell desmosomes, but not tight and adherens junctions, upon whipworm L1 larvae infection of mice and caecaloids.

Representative TEM images of *T. muris*-infected caecaloids and caecum after 72 h of infection showing: **a** Intact tight and adherens junctions (white stars) but separated desmosomes (red stars) on cells hosting the larva. Images are representative of observations in four and two worms in caecaloids and caecum, respectively; **b** Worm infecting IEC at the caecum (scale bar 3μm) and desmosomes (arrowheads) joining infected and adjacent cells from infected mice (inset) and cells from uninfected mice. Scale bars for desmosome images 100 nm. Desmosome separation in nm was measured in host cells from two worms. Adjacent measurements n=25, uninfected measurements n=25. Data are presented as median values with interquartile range. **p=0.0025 Mann-Whitney U two-tailed test. Source data are provided as a Source Data file. **c** Representative TEM images of desmosomes from uninfected and *T. muris*-infected caecaloids and caecum of mice. Scale bars 100 nm. Statistical comparison (Mann-Whitney U two-tailed test) between the desmosome separation measurements show no difference between the *in vitro* and *in vivo* models. Measurements: caecaloid uninfected n=50, caecum infected n=25, caecaloid infected n=62, caecum infected n=25. Data are presented as median values with interquartile range. Source data are provided as a Source Data file.

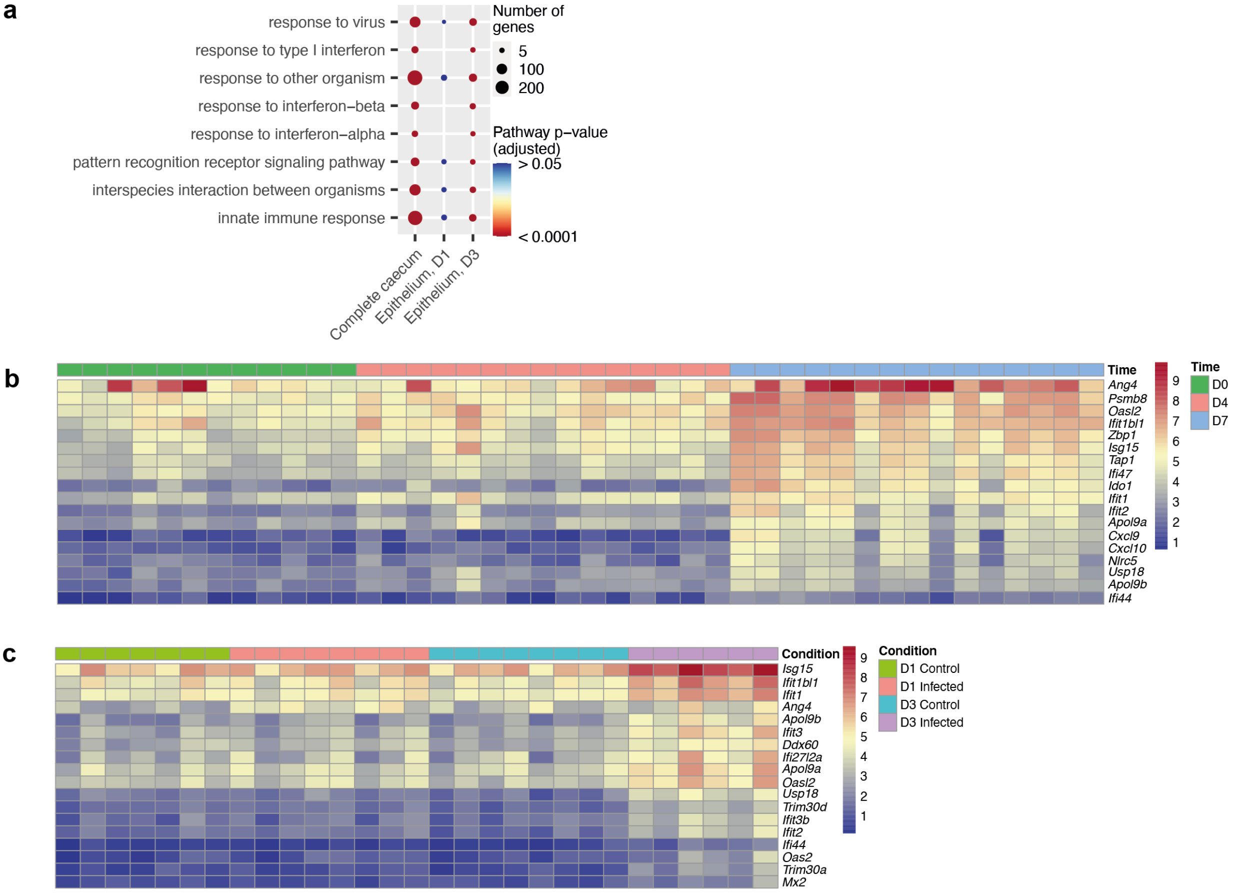

Supplementary Fig. 9. Host responses to early infection with whipworms are dominated by a type-I IFN signature.

a Selected significantly enriched gene ontology (GO) terms associated with differentially expressed genes in the complete caecum (day 7 post *T. muris* infection) and caecal IECs (days 1 and 3 post *T. muris* infection). The size of each circle corresponds to the numbers of genes annotated to a given term, while the colour indicates the FDR-adjusted p-value (n=4–5 mice per group). Overrepresented GO terms were identified with the goseq package (Wallenius method). **b** Heatmap (log2(FPKM+1)) of selected IFN-associated differentially expressed genes (all with log2FoldChange > 1.5 at 0 v 7 day p.i and FDR-adjusted p-value <0.05, likelihood ratio test) in the caecum of mice in response to *T. muris* infection at days 0 (control), 4 and 7 p.i (n=4–5 mice per group, with 3 caecum samples per mouse). **c** Heatmap (log2(FPKM+1)) of selected IFN-associated differentially expressed genes (all with log2FoldChange > 1.5 at day 3 p.i and FDR-adjusted p-value <0.05, Wald test) in caecal IECs at days 1 and 3 p.i, with time-matched controls (n=4 mice per group, with 1–2 technical replicates per mouse).

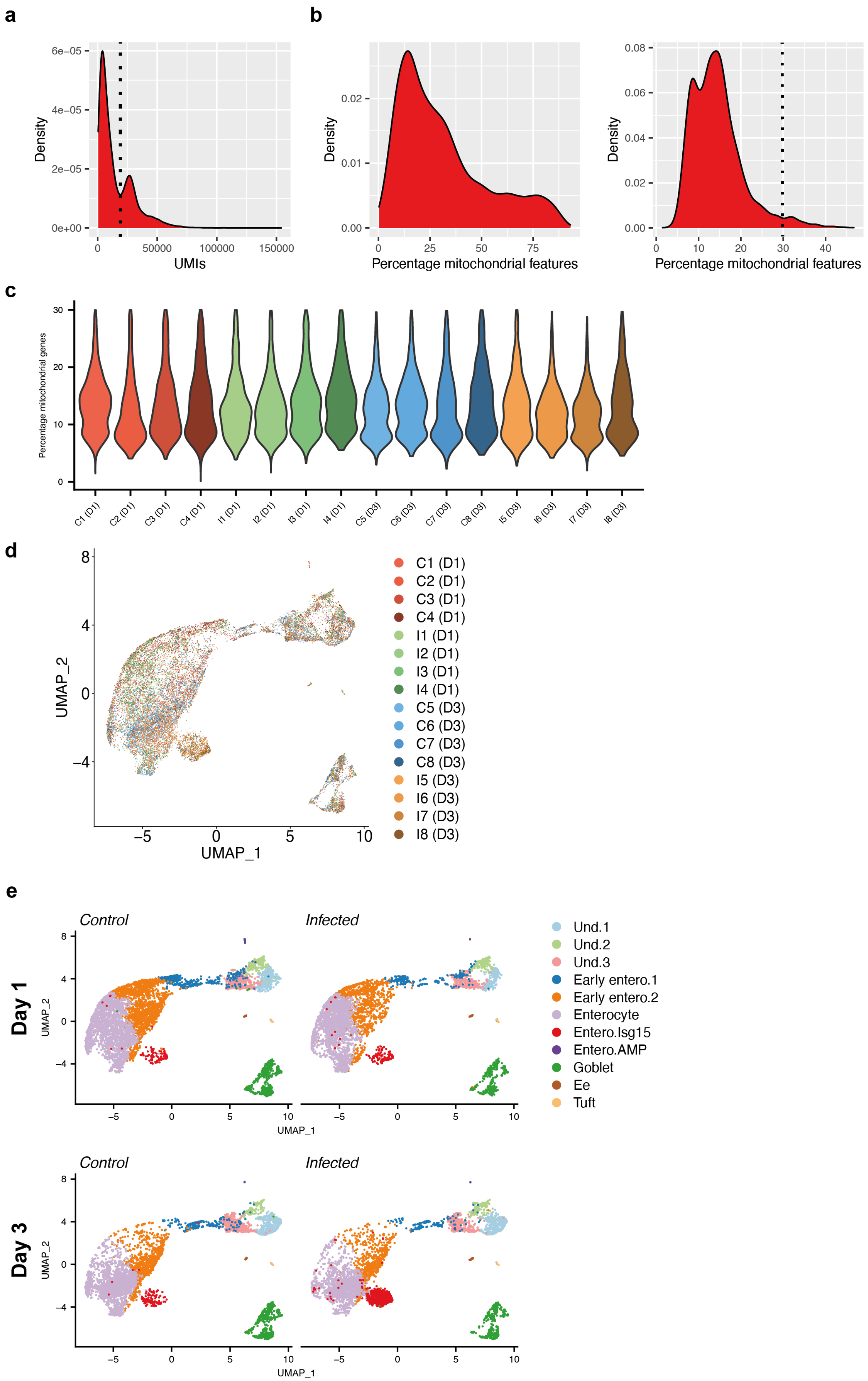
a
b
c
d
e
Density
UMIs
Percentage mitochondrial features
Percentage mitochondrial genes
UMAP_1
UMAP_2
C1 (D1)
C2 (D1)
C3 (D1)
C4 (D1)
I1 (D1)
I2 (D1)
I3 (D1)
I4 (D1)
C5 (D3)
C6 (D3)
C7 (D3)
C8 (D3)
I5 (D3)
I6 (D3)
I7 (D3)
I8 (D3)
Control
Infected
Day 1
Day 3
Und.1
Und.2
Und.3
Early entero.1
Early entero.2
Enterocyte
Entero.Isg15
Entero.AMP
Goblet
Ee
Tuft

119

Supplementary Fig 10. Single-cell RNA-seq data QC.

a Cellular barcodes in 10X data were selected above the first local minimum on a UMI density plot for each individual sample. Example distribution density and local minimum (dashed line) are shown. **b** Percentage of mitochondrial genes per cell for a representative sample pre (left panel) and post (right panel) UMI-based filtering. An additional filter at 30% mitochondrial genes per cell was then applied (dotted line). **c** Violin plots of percentage of mitochondrial genes per cell post QC (all samples; C= control, I=infected). **d** UMAP visualization of sample batch distribution in the merged clustering analysis (n=4 mice per group). **e** UMAP plots from single cell RNA-seq analysis of IEC populations (colour coded) in the caecum of control and *T. muris*-infected mice after 1 and 3 days p.i. (n=4 mice for each condition at each time point).

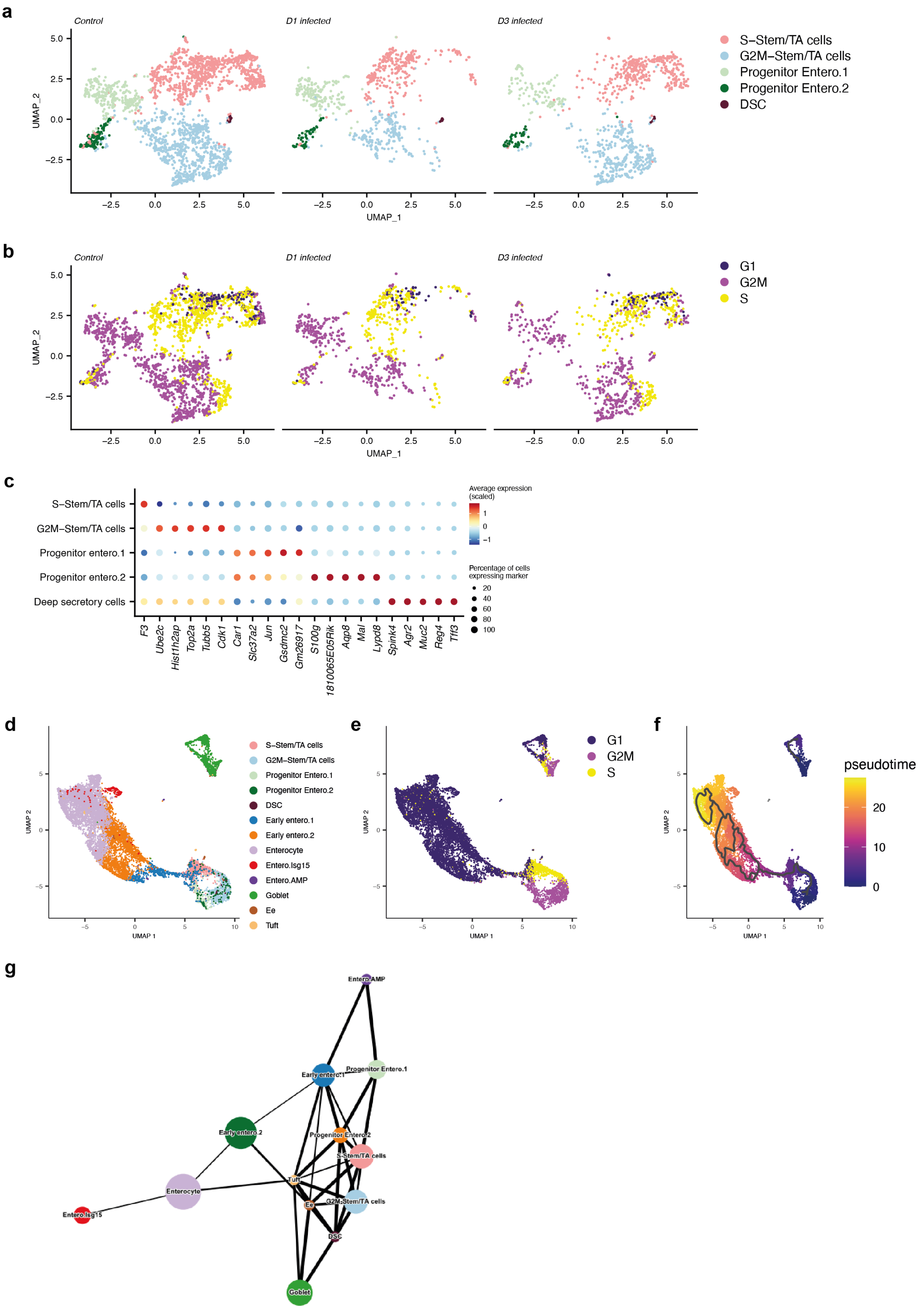
a
Control
D1 infected
D3 infected
UMAP_2
UMAP_1
S−Stem/TA cells
G2M−Stem/TA cells
Progenitor Entero.1
Progenitor Entero.2
DSC
b
G1
G2M
S
c
Average expression (scaled)
Percentage of cells expressing marker
S−Stem/TA cells
G2M−Stem/TA cells
Progenitor entero.1
Progenitor entero.2
Deep secretory cells
F3
Ube2c
Hist1h2ap
Top2a
Tubb5
Cdk1
Car1
Slc37a2
Jun
Gsdmc2
Gm26917
S100g
1810065E05Rik
Aqp8
Mal
Lypd8
Spink4
Agr2
Muc2
Reg4
Tff3
d
e
f
Early entero.1
Early entero.2
Enterocyte
Entero.Isg15
Entero.AMP
Goblet
Ee
Tuft
UMAP 1
UMAP 2
pseudotime
g
Progenitor Entero.1
Progenitor Entero.2
S-Stem/TA cells
G2M-Stem/TA cells
DSC

Supplementary Fig 11. *In silico* analysis of undifferentiated clusters.

a UMAP visualisation of subclustered undifferentiated cells, coloured by subcluster. **b** UMAP visualisation of undifferentiated cells, coloured by cell cycle phase**. c** Dot plot of the top marker genes for each cell type. The relative size of each dot represents the fraction of cells per cluster that expresses each marker; the colour represents the average (scaled) gene expression per cluster. **d** Monocle UMAP visualisation of all control cells, coloured by cluster (integrating subclustering of undifferentiated cells). **e** Monocle UMAP visualisation of all control cells, coloured by cell cycle phase. **f** Monocle UMAP visualisation of all control cells, coloured by pseudotime. **g** Partition-based graph abstraction (PAGA) visualisation of all control cells. Line thickness represents relatedness between clusters.

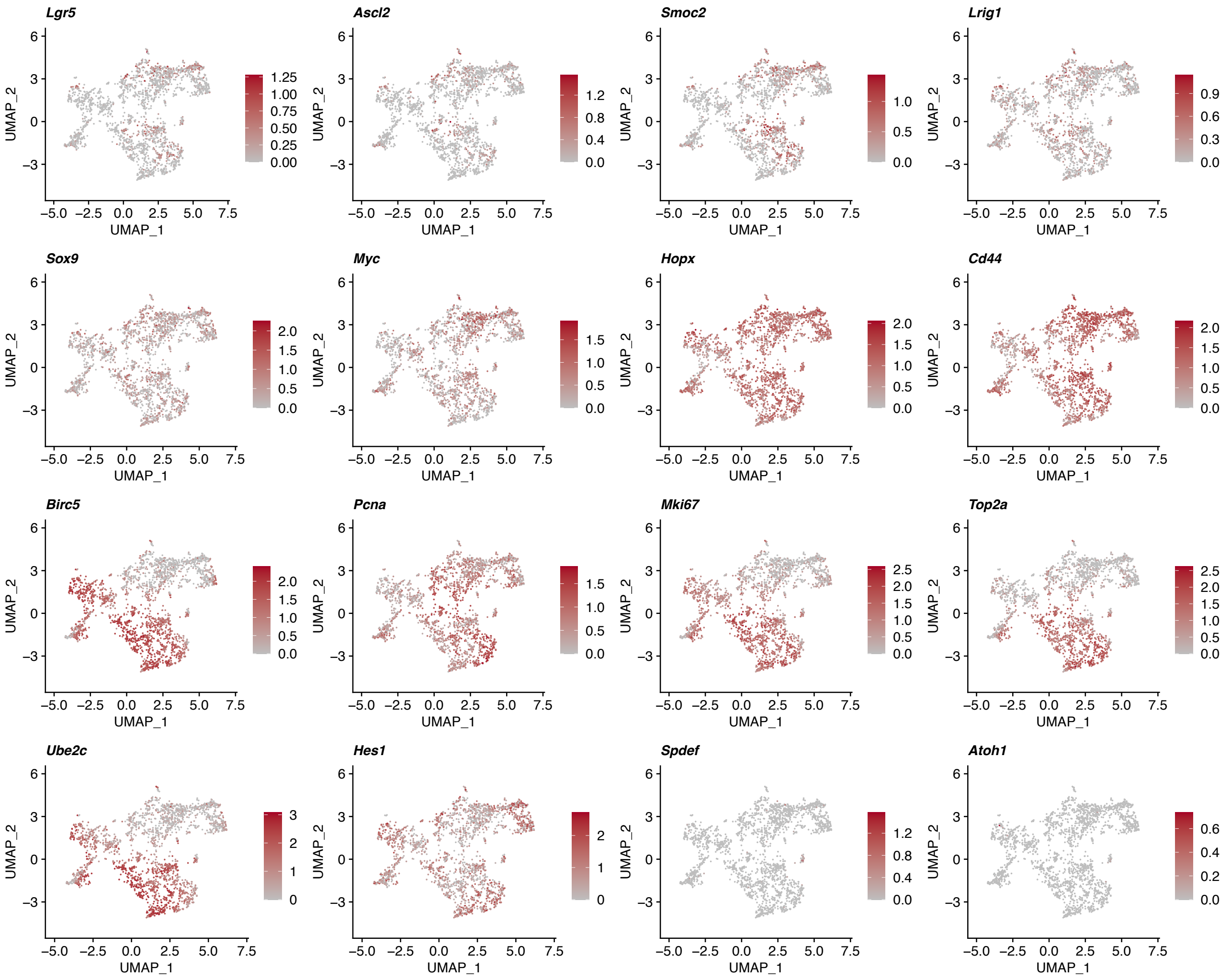

Supplementary Fig 12. Expression of stem, TA and progenitor cell markers by IECs from the caecum of uninfected mice.

UMAP plots from single cell RNA-seq analysis showing normalized expression of *Lgr5*, *Ascl2, Smoc2, Lrig1*, *Sox9*, *Myc*, *Hopx, Cd44, Birc5, Pcna, Mki67, Top2a, Ubec2c, Hes1, Spdef* and *Atoh1* in IECs from the caecum of control mice (n=8).

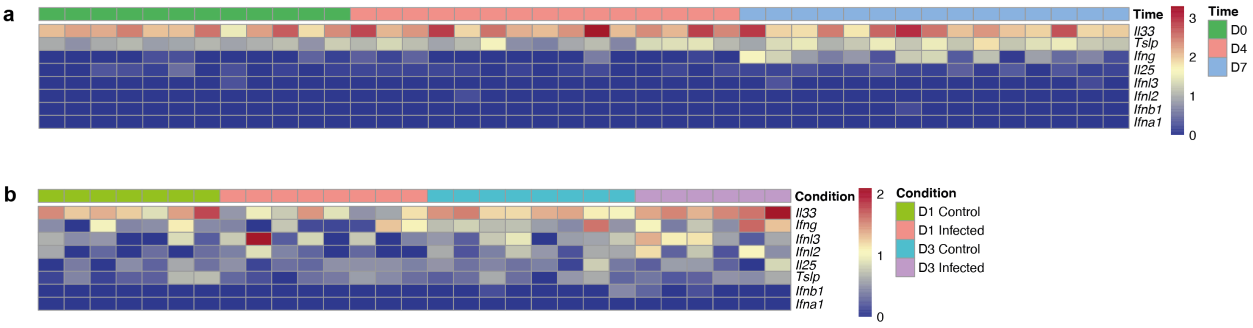

Supplementary Fig 13. Expression of cytokines and alarmins in the caecum and caecal IECs of uninfected and *T. muris*-infected mice upon early infection.

a Expression (log2(FPKM+1)) of *Il33*, *Tslp*, *Ifng, Il25*, *Ifnl3, Ifnl2, Ifnb1* and *Ifna1* in the caecum of mice at days 0, 4 and 7 post *T. muris* infection (n=4-5 mice per group, with 3 caecum samples per mouse). **b** Expression (log2(FPKM+1)) of *Il-33*, *Ifng, Ifnl3, Ifnl2, Il-25*, *Tslp, Ifnb1* and *Ifna1* in sorted murine caecal IECs in response to *T. muris* infection at days 1 and 3 p.i (n=4 mice per group, with 1-2 technical replicates per mouse).

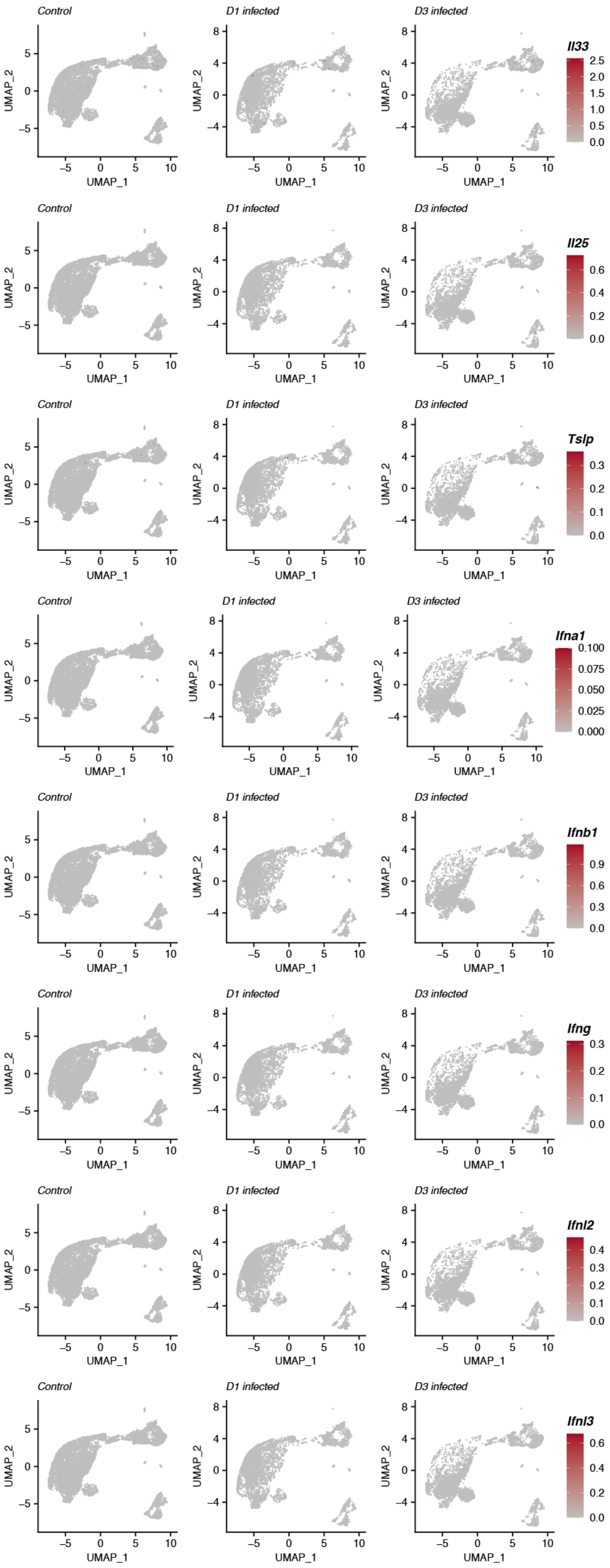

Supplementary Fig 14. Expression of cytokines and alarmins by IECs from the caecum of uninfected and *T. muris*-infected mice upon early infection.

UMAP plots from single cell RNA-seq analysis showing normalized expression levels of *Il33*, *Il25*, *Tslp*, *Ifna1*, *Ifnb1, Ifng, Ifnl2* and *Ifnl3* in IECs from the caecum of control (n=8, 4 mice for each time point) and *T. muris*-infected mice after 1 and 3 days p.i. (n=4 mice for each time point).
