# Supplementary material for: Defining the early stages of intestinal colonisation by whipworms
Source: Nat Commun. 2022 Apr 1;13:1725. doi: 10.1038/s41467-022-29334-0 (PMC8976045; doi:10.1038/s41467-022-29334-0)
Supplement: Supplementary file 3 — Description of Additional Supplementary Files [file 41467_2022_29334_MOESM3_ESM.pdf]

File Name: Supplementary Data 1

Description: Differentially regulated genes and enriched GO (BP) terms in whole caecum of mice across a seven-day T. muris infection time course.

File Name: Supplementary Data 2

Description: Differentially regulated genes and enriched GO (BP) terms in caecal IECs at day 1 and 3 post T. muris infection.

File Name: Supplementary Data 3

Description: Cluster marker genes and enriched GO (BP) terms for caecal IEC populations identified by single-cell RNAseq.

File Name: Supplementary Movie 1

Description: Movie visualising serial block face SEM images of syncytial tunnel burrowed by a L1 larva in the caecum of a *T. muris*-infected mouse at 24 h p.i.

File Name: Supplementary Movie 2

Description: Movie visualising serial block face SEM images of syncytial tunnel burrowed by another *T. muris* L1 larva in the caecum of a *T. muris*-infected mouse at 24 h p.i.

File Name: Supplementary Movie 3

Description: Movie visualising z-stack of confocal IF images of caecaloids infected with *T. muris* L1 larvae for 24 h. Intricate tunnels are left behind by larva, which are completely multi-intracellular. Nuclei of both the epithelia cells and larvae are (DAPI) stained blue, F-actin at cell membrane (phalloidin) is white, mucus vacuoles of goblet cells (UEA/SNA lectins binding mucin glycans) are green and dividing cells (Ki-67) are magenta.

File Name: Supplementary Movie 4

Description: Movie visualising z-stack of confocal IF images of *T. muris*-infected caecaloids (24 h p.i.). Intricate tunnels are left behind by larva, which are completely multi-intracellular. Nuclei of both the epithelia cells and larvae are (DAPI) stained blue, F-actin at cell membrane (phalloidin) is white, mucus vacuoles of goblet cells (UEA/SNA lectins binding mucin glycans) are green and tuft cells (Dckl-1) are red.

File Name: Supplementary Movie 5

Description: Movie visualising z-stack of confocal IF images of tight junctions of host IECs from *T. muris* L1 larva in syncytial tunnel in caecaloids (24 h p.i.). Tight junctions of infected IECs are preserved. Nuclei of both the epithelia cells and larvae are (DAPI) stained blue, F-actin at cell membrane (phalloidin) is white, mucus vacuoles of goblet cells (UEA/SNA lectins binding mucin glycans) are green and tight junctions (ZO-1 protein) are red.
