# Supplementary material for: Defining the early stages of intestinal colonisation by whipworms
Source: Nat Commun. 2022 Apr 1;13:1725. doi: 10.1038/s41467-022-29334-0 (PMC8976045; doi:10.1038/s41467-022-29334-0)
Supplement: Supplementary file 12 — Reporting Summary [file 41467_2022_29334_MOESM12_ESM.pdf]

nature portfolio

Corresponding author(s): Matthew Berriman & Maria A. Duque-Correa

Last updated by author(s): 14-02-2022

Reporting Summary

Nature Portfolio wishes to improve the reproducibility of the work that we publish. This form provides structure for consistency and transparency in reporting. For further information on Nature Portfolio policies, see our Editorial Policies and the Editorial Policy Checklist.

Statistics

For all statistical analyses, confirm that the following items are present in the figure legend, table legend, main text, or Methods section.

n/a	Confirmed	
☐	☒	The exact sample size (*n*) for each experimental group/condition, given as a discrete number and unit of measurement
☐	☒	A statement on whether measurements were taken from distinct samples or whether the same sample was measured repeatedly
☐	☒	The statistical test(s) used AND whether they are one- or two-sided<br>*Only common tests should be described solely by name; describe more complex techniques in the Methods section.*
☐	☒	A description of all covariates tested
☐	☒	A description of any assumptions or corrections, such as tests of normality and adjustment for multiple comparisons
☐	☒	A full description of the statistical parameters including central tendency (e.g. means) or other basic estimates (e.g. regression coefficient) AND variation (e.g. standard deviation) or associated estimates of uncertainty (e.g. confidence intervals)
☐	☒	For null hypothesis testing, the test statistic (e.g. *F*, *t*, *r*) with confidence intervals, effect sizes, degrees of freedom and *P* value noted<br>*Give P values as exact values whenever suitable.*
☒	☐	For Bayesian analysis, information on the choice of priors and Markov chain Monte Carlo settings
☒	☐	For hierarchical and complex designs, identification of the appropriate level for tests and full reporting of outcomes
☒	☐	Estimates of effect sizes (e.g. Cohen's *d*, Pearson's *r*), indicating how they were calculated

Our web collection on statistics for biologists contains articles on many of the points above.

Software and code

Policy information about availability of computer code

Data collection	No software was used
Data analysis	Prism 9 software (GraphPad) was used for Mann-Whitney U two-tailed and Kruskal Wallis and Dunn's comparison tests.<br>For bulk and single-cell RNA-seq data analysis, Kallisto (v0.42.3, v0.46.2), R (v3.6.1), CellRanger software suite (v3.0.2) and Python software (v3.7) were used. The main R packages used are: , DESeq2 (v1.22.2 and v1.26.0), TopGO (v2.34.0), pheatmap (v1.0.11), GOseq (v1.38.0), fgsea (v1.12.0), Seurat package (v3.0.2), DoubletDecon (v1.1.4) and monocle3 (v0.2.1). In Python, the package SCANPY was used (v 1.4.6.).<br><br>The R code used to analyse the bulk and single-cell RNA-seq data of this study is available in the Github repository https://github.com/fayerodgers/single_cell, DOI 10.5281/zenodo.598409282.

For manuscripts utilizing custom algorithms or software that are central to the research but not yet described in published literature, software must be made available to editors and reviewers. We strongly encourage code deposition in a community repository (e.g. GitHub). See the Nature Portfolio guidelines for submitting code & software for further information.

Data

Policy information about availability of data

All manuscripts must include a data availability statement. This statement should provide the following information, where applicable:

- Accession codes, unique identifiers, or web links for publicly available datasets
- A description of any restrictions on data availability
- For clinical datasets or third party data, please ensure that the statement adheres to our policy

All the data needed to make the conclusions from this study are present in the main manuscript or supplementary materials. Source data are provided with this

paper. The transcriptomic datasets generated during and analysed the current study are available in the European Nucleotide Archive (ENA) repository (https://www.ebi.ac.uk/ena/browser/home), under the accession numbers ERP008531 (STDY 3371 - Mouse and Trichuris muris transcriptome time course, https://www.ebi.ac.uk/ena/browser/view/PRJEB7610?show=reads), ERP126662 (STDY 4023 - Investigating the transcriptome of early infective larvae stage of Trichuris muris, https://www.ebi.ac.uk/ena/browser/view/PRJEB42759?show=reads) and ERP021944 (STDY 4672 - Investigating early host intestinal epithelial cells - whipworm interactions, https://www.ebi.ac.uk/ena/browser/view/PRJEB19877?show=reads). Databases used for the analysis are : Ensembl (https://www.ensembl.org/index.html), the molecular signatures database (http://www.gsea-msigdb.org/gsea/msigdb/) and WormBase Parasite (https://parasite.wormbase.org/index.html).

Field-specific reporting

Please select the one below that is the best fit for your research. If you are not sure, read the appropriate sections before making your selection.

☒ Life sciences ☐ Behavioural & social sciences ☐ Ecological, evolutionary & environmental sciences

For a reference copy of the document with all sections, see nature.com/documents/nr-reporting-summary-flat.pdf

Life sciences study design

All studies must disclose on these points even when the disclosure is negative.

Sample size	Sample size for bulk and single-cell RNAseq experiment was determined by performing power calculations.<br>Imaging experiments were qualitative, therefore no power calculations were done to estimate the sample size, which reflect the number of whipworm larvae found in the caecum of mice (after extensive sectioning) or caecaloids. However, to account for biological variance, in in vivo experiments three mice per group were evaluated, and in vitro experiments were performed using caecaloid lines from 3 different mice, repeated at least 2 independent times and including each time a minimum of 3 technical replicates per line per condition (time point and infection status) to ensure reproducibility. In these studies, the experimental unit is not the mouse or the caecaloid line but either the whipworm larvae, cell types or desmosomes for which we have included sample sizes higher than 10.
Data exclusions	No data were excluded from the results presented in this manuscript.
Replication	At least two independent experiments using 2-3 caecaloid lines derived from independent mice were carried out to ensure reproducibility for in vitro experiments. For in vivo experiments, at least two independent experiments were carried out. All attempts of replication were successful.
Randomization	For in vitro experiments, caecaloids are grown in 6-well transwell plates, which were randomly allocated into uninfected and infected groups at the moment of infection in order to have 3 replicates per condition. For in vivo experiments, mice were randomly allocated into uninfected and infected groups using the Graph Pad Prism randomization tool.
Blinding	In vitro experiments were not carried out with blinding as for the majority of the experiments the same researcher was performing the infection and measuring the readouts, but at least two independent repeats were performed. For in vivo experiments, blinding at the point of measurement was achieved using barcodes. During sample collection, group membership could be seen, however this stage was completed by technician staff with no knowledge of the experiment objectives. For SEM and TEM imaging experiments, blinding is not possible as the researcher performing them is looking for microscopic larvae infecting less than 1% of the caecal epithelia and therefore needs to screen hundreds of sections.

Reporting for specific materials, systems and methods

We require information from authors about some types of materials, experimental systems and methods used in many studies. Here, indicate whether each material, system or method listed is relevant to your study. If you are not sure if a list item applies to your research, read the appropriate section before selecting a response.

Materials & experimental systems

n/a	Involved in the study	
☐	☒	Antibodies
☒	☐	Eukaryotic cell lines
☒	☐	Palaeontology and archaeology
☐	☒	Animals and other organisms
☒	☐	Human research participants
☒	☐	Clinical data
☒	☐	Dual use research of concern

Methods

n/a	Involved in the study	
☒	☐	ChIP-seq
☒	☐	Flow cytometry
☒	☐	MRI-based neuroimaging

Antibodies

Antibodies used	rabbit α-p43 primary antibody (Cambridge Research Biochemicals - not commercially available, 1:400); α-villin (Abcam, ab130751, clone SP145, 1:100); α-Ki-67 (Abcam, ab16667, clone SP6, 1:250); α-chromogranin A (Abcam, ab15160, 1:50); α-Dcamlk-1 (Abcam, ab31704, 1:200); α-zona occludens-1 (ZO-1) protein (Invitrogen, 61-7300, 1:200); donkey anti-rabbit IgG Alexa Fluor 555 (Molecular

	Probes, A31572, 1:400); AF488-conjugated goat-anti-rabbit IgG (Invitrogen, A11008, 1:500); α-CD236 (epithelial cell adhesion molecule (EPCAM); PE-Cy7, Biolegend, 118216, clone G8.8, 1:300); α-CD45 (Alexa 700, Biolegend, 103128, clone 30-F11, 1:300); and rabbit α-MUC2 primary antibody (MAN-2I, generated by Prof. David J. Thornton - not commercially available, 1:1000).
Validation	The rabbit α-p43 (Cambridge Research Biochemicals) was validated by Bancroft, A.J., Levy, C.W., Jowitt, T.A. et al. The major secreted protein of the whipworm parasite tethers to matrix and inhibits interleukin-13 function. Nat Commun 10, 2344 (2019). https://doi.org/10.1038/s41467-019-09996-z.<br><br>The rabbit α-MUC2 primary antibody (MAN-2I) is a polyclonal antiserum raised against a synthetic peptide, NGLQPVRVEDPDGC, in the C-terminal region of MUC2 as previously described for the MUC2 probe LUM2-3 (Carlstedt, I. et al. 'Soluble' and 'insoluble' mucins--identification of distinct populations. Biochem Soc Trans 23, 845-851, doi:10.1042/bst0230845 (1995).https://doi.org/10.1042/bst0230845). Prof Thornton has validated the MAN-2I antibody and used it in several publications:<br>- Thornton, D. J. et al. Characterization of mucins from cultured normal human tracheobronchial epithelial cells. Am J Physiol Lung Cell Mol Physiol 278, L1118-1128, doi:10.1152/ajplung.2000.278.6.L1118 (2000).<br>- Kirkham, S., Sheehan, J. K., Knight, D., Richardson, P. S. & Thornton, D. J. Heterogeneity of airways mucus: variations in the amounts and glycoforms of the major oligomeric mucins MUC5AC and MUC5B. Biochem J 361, 537-546, doi:10.1042/0264-6021:3610537 (2002).<br>- Kirkham, S. et al. MUC5B is the major mucin in the gel phase of sputum in chronic obstructive pulmonary disease. Am J Respir Crit Care Med 178, 1033-1039, doi:10.1164/rccm.200803-391OC (2008).<br><br>Validation for commercial antibodies available at:<br>https://www.abcam.com/villin-antibody-sp145-ab130751.html, https://www.abcam.com/ki67-antibody-sp6-ab16667.html, https://www.abcam.com/chromogranin-a-antibody-ab15160.html, https://www.abcam.com/dcamkl1-antibody-ab31704.html, https://www.thermofisher.com/antibody/product/ZO-1-Antibody-Polyclonal/61-7300, https://www.thermofisher.com/antibody/product/Donkey-anti-Rabbit-IgG-H-L-Highly-Cross-Adsorbed-Secondary-Antibody-Polyclonal/A-31572, https://www.thermofisher.com/antibody/product/Goat-anti-Rabbit-IgG-H-L-Cross-Adsorbed-Secondary-Antibody-Polyclonal/A-11008, https://www.biolegend.com/en-us/products/pe-cyanine7-anti-mouse-cd326-ep-cam-antibody-5303?GroupID=BLG6455, and https://www.biolegend.com/en-us/products/alexa-fluor-700-anti-mouse-cd45-antibody-3407?GroupID=BLG6833.

Animals and other organisms

Policy information about studies involving animals; ARRIVE guidelines recommended for reporting animal research

Laboratory animals	Mus musculus. C57BL/6N, female 6-8 week old mice.
Wild animals	No wild animals were used in this study.
Field-collected samples	No field collected samples were used in this study.
Ethics oversight	Experiments were performed under the regulation of the UK Animals Scientific Procedures Act 1986 under the Project licenses 80/2596 and P77E8A062, and were approved by the Wellcome Sanger Institute Animal Welfare and Ethical Review Body and followed ARRIVE guidelines.

Note that full information on the approval of the study protocol must also be provided in the manuscript.
